# Gestational Age–Adapted Laparoscopic Appendectomy for Acute Appendicitis during Pregnancy: Intraoperative Strategies and Outcomes from a Single-Center Case Series

**DOI:** 10.70352/scrj.cr.26-0420

**Published:** 2026-07-24

**Authors:** Yusuke Kitani, Takamune Yamaguchi, Kosuke Nakane, Takayoshi Koseki, Koichiro Kawasaki, Michiro Takahashi, Kentaro Inada

**Affiliations:** Department of Surgery, Tokyo Metropolitan Bokutoh Hospital, Tokyo, Japan

**Keywords:** acute appendicitis, pregnancy, laparoscopic appendectomy, pneumoperitoneum pressure, patient positioning

## Abstract

**INTRODUCTION:**

Acute appendicitis is the most common non-obstetric surgical emergency during pregnancy, occurring in approximately 1 in 1000–2000 pregnancies. Although laparoscopic appendectomy (LA) offers advantages over open appendectomy, including reduced postoperative pain and a shorter hospital stay, its application in pregnant patients remains debated due to concerns about fetal effects and the technical challenges posed by the enlarged uterus.

**CASE PRESENTATION:**

We retrospectively reviewed 6 consecutive pregnant women who underwent LA for acute appendicitis at our institution between April 2020 and April 2025, focusing on intraoperative techniques and surgical outcomes. Patient age ranged from 28 to 37 years (median, 31.5 years) and gestational age from 15 to 33 weeks (median, 21.0 weeks). All cases were completed laparoscopically using a 3-port technique, with intraperitoneal insufflation pressure maintained at 10–12 mmHg. A supine position was used in 4 cases (≤20 weeks of gestation) and a left lateral decubitus position in 2 cases (≥21 weeks). The median operative time was 84 min (range, 64–124 min), blood loss was 0 g in 5 cases (5 g in 1), and the median postoperative hospital stay was 5 days (range, 2–7 days). Pathological findings included 1 catarrhal, 4 phlegmonous, and 1 gangrenous appendicitis. No conversions to open surgery occurred, and no obstetric complications were observed in any case.

**CONCLUSIONS:**

By tailoring patient positioning and port placement to gestational age, LA was safely performed during the 2nd and 3rd trimesters. LA appears to be a safe and effective treatment for acute appendicitis during pregnancy when appropriate intraoperative precautions are applied.

## Abbreviations

EAES
: European Association for Endoscopic Surgery
EtCO_2_
: end-tidal carbon dioxide
LA
: laparoscopic appendectomy
OA
: open appendectomy
SAGES
: Society of American Gastrointestinal and Endoscopic Surgeons

## INTRODUCTION

Acute appendicitis is the most common non-obstetric cause of acute abdomen during pregnancy, with an estimated incidence of approximately 1 in 1000–2000 pregnancies.^1)^ Displacement of the appendix superiorly and to the right by the enlarging uterus, combined with nausea and vomiting attributable to pregnancy itself, can make early diagnosis challenging.^2)^ Delayed diagnosis increases the risk of perforation and peritonitis, which may lead to serious obstetric complications, including preterm birth and fetal loss.^3)^

Surgical intervention remains the mainstay of treatment. In nonpregnant patients, LA has become the standard of care, demonstrating superiority over OA in terms of postoperative pain, wound infection, hospital stay, time to recovery, and cosmesis.^4)^ However, the optimal surgical approach for pregnant patients with acute appendicitis has been debated for decades. The SAGES 2024 updated guidelines conditionally recommend LA regardless of gestational age.^5)^ Specifically, LA is weakly recommended when the uterine fundus is at or below the level of the umbilicus; when the fundus is above the umbilicus, either LA or OA may be selected based on the surgeon’s experience and skill. The open (Hasson) technique is recommended for establishing pneumoperitoneum.^5)^ The EAES 2022 guideline weakly recommends LA with an open-entry technique when the gestational age is less than 20 weeks or the uterine fundus is at or below the umbilicus and suggests that either LA or OA may be selected based on the surgeon’s expertise beyond 20 weeks.^6)^ A key concern regarding LA in pregnancy is the potential fetal effect of CO_2_ pneumoperitoneum. An animal study demonstrated that insufflation pressures exceeding 15 mmHg induced fetal acidosis in a pregnant ewe model^7)^; however, adverse fetal events attributable to CO_2_ pneumoperitoneum have not been confirmed in humans.^5)^

In Japan, no standardized national guidelines exist for the management of appendicitis during pregnancy, and institutional approaches vary widely. Nakashima et al. reported that the nationwide LA utilization rate for appendicitis in pregnant women remains as low as 8%.^8)^ Against this background, we present our single-center experience with LA in 6 pregnant women, with a focus on intraoperative techniques and safety outcomes.

## CASE PRESENTATION

### Methods

#### Study design and patients

This retrospective study included 6 consecutive pregnant women who underwent LA for acute appendicitis at the Department of Surgery, Tokyo Metropolitan Bokutoh Hospital, between April 2020 and April 2025. Patient demographics, preoperative laboratory and imaging findings, intraoperative records, postoperative course, and obstetric outcomes were collected from medical records.

#### Diagnosis

The diagnosis of acute appendicitis was established based on a combination of clinical findings (right lower quadrant pain, fever, and rebound tenderness), laboratory data (leukocyte count and C-reactive protein), and imaging studies (abdominal US, CT, or MRI when necessary). CT was performed only when essential for diagnosis, with careful consideration of fetal radiation exposure.

#### Surgical technique

**Fig. 1:**
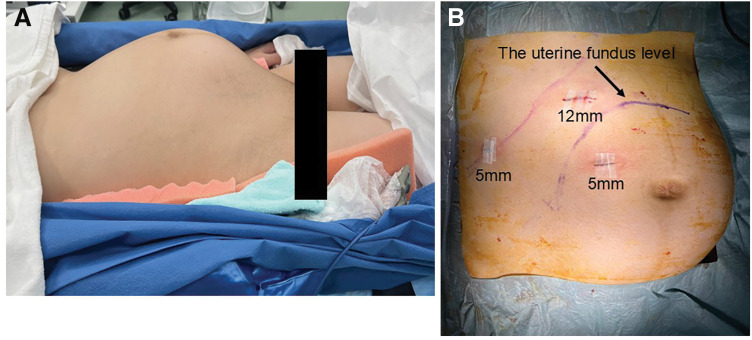
Intraoperative positioning and port placement in Case 5 (33 weeks of gestation). (**A**) A triangular pillow was placed under the right lumbar region to achieve a left lateral decubitus position; a positioning mat was used to facilitate intraoperative repositioning. Note: The positioning mat is not clearly visible in the photograph due to surgical draping, but was placed beneath the patient prior to the procedure to enable position adjustment if required. (**B**) The uterine fundus level was identified and marked on the abdominal surface based on preoperative CT. A 12-mm camera port was inserted in the right subcostal region using the open (Hasson) technique, and 2 additional 5-mm working ports were placed in a coaxial configuration.

**Fig. 2:**
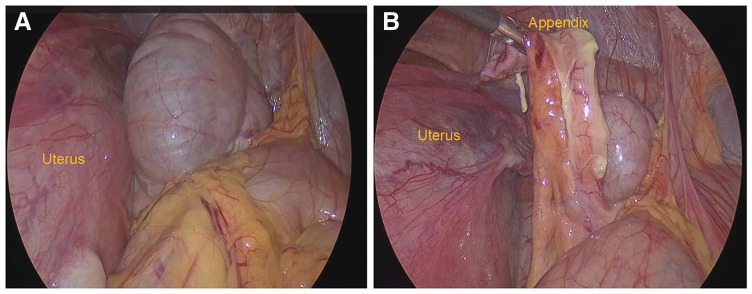
Laparoscopic findings in Case 5 (viewed from the right subcostal camera port). (**A**) The uterine fundus was located above the umbilicus; however, the left lateral decubitus position provided adequate displacement of the uterus and bowel to the left, ensuring a satisfactory operative view. (**B**) Identification and elevation of the appendix were achieved without difficulty.

**Fig. 3:**
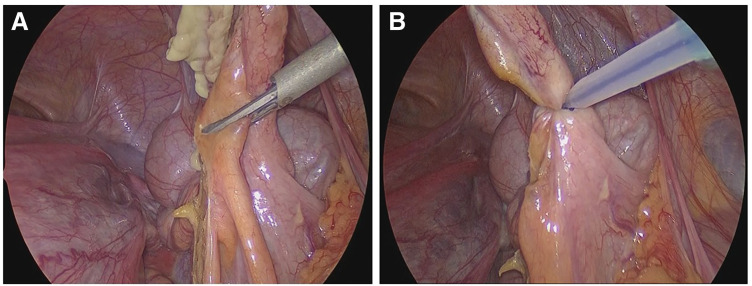
Laparoscopic findings in Case 5 (continued). (**A**) Coaxial trocar placement enabled mesoappendix dissection to be performed safely without instrument interference from the enlarged uterus. (**B**) Appendiceal base ligation was completed safely without instrument interference from the enlarged uterus.

All procedures were performed under general anesthesia using a 3-port technique. Preoperative CT was used to identify the position of the uterine fundus and guide individual port placement. Pneumoperitoneum was established using the open (Hasson) technique. Insufflation pressure was maintained at 10 mmHg as a standard, with a maximum of 12 mmHg when necessary. Port placement was tailored according to the gestational age and the preoperatively confirmed uterine fundus level. For cases at ≤20 weeks of gestation (with the uterine fundus at or below the umbilical level), patients were placed in the supine position, and a conventional 3-port configuration was used: a 12-mm camera port was inserted at the umbilicus using the open (Hasson) technique, and two 5-mm working ports were placed in the right iliac fossa and the right lateral abdomen in a coaxial arrangement. For cases at ≥21 weeks of gestation (uterine fundus above the umbilical level), a triangular pillow was placed under the right lumbar region to achieve a left lateral decubitus position; a positioning mat was used to facilitate intraoperative repositioning if required. In these cases, a 12-mm camera port was inserted into the right subcostal region using the open (Hasson) technique, and 2 additional 5-mm working ports were placed inferiorly in a coaxial configuration based on the CT-confirmed fundal position. Continuous capnographic monitoring of EtCO_2_ was performed throughout the procedure in close collaboration with the anesthesiologist. Fetal heart rate monitoring was performed by an obstetrician both before and after surgery in all cases. Perioperative prophylactic antibiotics (1st- or 2nd-generation cephalosporins) were administered in accordance with institutional protocol. Tocolytic agents were not administered in any case; uterine contractility was monitored postoperatively by the obstetric team in all cases. Intraoperative positioning, port placement, and laparoscopic findings for Case 5 (33 weeks of gestation) are illustrated in **Figs. 1**–**3**.

#### Outcome measures

The primary outcome measure was obstetric complications, defined as preterm birth, spontaneous abortion, or fetal distress. Secondary outcomes included operative time, blood loss, conversion rate to OA, postoperative hospital stay, postoperative complications, and histopathological findings.

### Results

#### Patient characteristics

The clinical characteristics and surgical outcomes of all 6 cases are summarized in **Table 1**. Patient age ranged from 28 to 37 years (median, 31.5 years). Gestational age at the time of surgery ranged from 15 to 33 weeks (median, 21.0 weeks): no cases were in the 1st trimester, 5 were in the 2nd trimester (13–27 weeks), and 1 was in the 3rd trimester (28 weeks or beyond). Leukocytosis (12000–17400/μL) was observed in all patients. Abdominal CT demonstrated appendiceal enlargement (8–13 mm in diameter) with periappendiceal fat stranding in all cases, confirming the diagnosis of acute appendicitis.

**Table 1:** Clinical characteristics and surgical outcomes of 6 pregnant patients who underwent LA

Case	Age (years)	Gestational age (weeks)	Intraoperative position	Ports (n)	IAP (mmHg)	EBL (g)	Operative time (min)	LOS (days)	Obstetric outcome	Pathology
1	36	22	Left lateral decubitus	3	12	0	124	7	Delivered at 39 wk	Catarrhal
2	33	23	Supine	3	10	0	99	7	N/A	Phlegmonous
3	28	20	Supine	3	10	0	77	4	N/A	Phlegmonous
4	37	15	Supine	3	10	5	72	6	N/A	Gangrenous
5	30	33	Left lateral decubitus	3	10	0	64	3	Delivered at 39 wk	Phlegmonous
6	28	20	Supine	3	10	0	91	2	Delivered at 37 wk	Phlegmonous

Intraoperative position: left lateral decubitus was used for cases at ≥21 weeks of gestation; supine position was used for cases at ≤20 weeks. N/A: obstetric follow-up data were not available and delivery outcomes could not be confirmed.

EBL, estimated blood loss; IAP, intraabdominal pressure; LA, laparoscopic appendectomy; LOS, length of postoperative hospital stay; N/A, not available; wk, weeks of gestation

#### Surgical outcomes

LA was completed in all 6 cases without conversion to open surgery. A left lateral decubitus position was used in 2 cases at ≥21 weeks of gestation (Cases 1 and 5), while the remaining 4 cases at ≤20 weeks of gestation were operated on in the supine position. For cases in the supine position, a conventional 3-port configuration was used: a 12-mm umbilical camera port with two 5-mm working ports in the right iliac fossa and right lateral abdomen in a coaxial arrangement. For cases in the left lateral decubitus position, a 12-mm right subcostal camera port with two 5-mm working ports was placed inferiorly in a coaxial configuration based on the CT-confirmed uterine fundus position. Insufflation pressure was 12 mmHg in Case 1 and 10 mmHg in the remaining 5 cases. Operative time ranged from 64 to 124 min (median, 84 min). Blood loss was 0 g in 5 cases, with 1 case (Case 4) recording 5 g of blood loss. Postoperative hospital stay ranged from 2 to 7 days (median, 5 days). Histopathological examination revealed catarrhal appendicitis in 1 case, phlegmonous appendicitis in 4, and gangrenous appendicitis in 1. No cases of perforated appendicitis or abscess formation were identified.

#### Obstetric outcomes

No obstetric complications—including preterm birth, spontaneous abortion, or fetal distress—were observed in any of the 6 cases. Case 1 (operated on at 22 weeks) continued the pregnancy uneventfully and delivered vaginally at 39 weeks. Case 5 (operated on at 33 weeks) also maintained the pregnancy without obstetric complications and achieved vaginal delivery at 39 weeks. Case 6 (operated on at 20 weeks) delivered at 37 weeks’ gestation. Obstetric follow-up data were not available for Cases 2, 3, and 4, and delivery outcomes for these patients could not be confirmed (**Table 1**).

## DISCUSSION

### International trends in LA for appendicitis during pregnancy

The optimal surgical approach for appendicitis during pregnancy has been debated for decades. Several systematic reviews and meta-analyses have consistently demonstrated that LA is associated with a shorter postoperative hospital stay without an increase in obstetric adverse events compared with OA.^4,9)^ As detailed in the Introduction, current SAGES and EAES guidelines conditionally recommend LA—with open-entry pneumoperitoneum—across gestational ages, with the surgical approach for advanced pregnancies individualized according to the surgeon’s expertise.^5,6)^ An important caveat in interpreting these recommendations is that the observed higher rate of fetal loss in the LA group in some earlier meta-analyses may reflect confounding by gestational age, as LA tends to be performed more frequently in the 1st trimester, during which spontaneous abortion rates are inherently higher.^6,9)^

In Japan, nationwide utilization of LA for appendicitis in pregnant women remains low. Nakashima et al. reported, using a nationwide administrative claims database, that only 8% of appendectomies in pregnant women were performed laparoscopically.^8)^ More recently, a multicenter collaborative study by Ogawa et al. demonstrated that both the LA and OA groups achieved zero rates of fetal loss, with a significantly shorter postoperative hospital stay in the LA group,^10)^ further supporting the safety and efficacy of LA in the Japanese population.

### Pneumoperitoneum pressure and fetal effects

The potential adverse effects of CO_2_ pneumoperitoneum on the fetus represent a key concern in performing LA during pregnancy. In a landmark animal study, Hunter et al. demonstrated that insufflation pressures exceeding 15 mmHg induced respiratory acidosis in fetal lambs.^7)^ Although adverse fetal events attributable to CO_2_ pneumoperitoneum have not been confirmed in humans,^5)^ SAGES recommends maintaining intraperitoneal pressure at or below 15 mmHg and performing continuous capnographic EtCO_2_ monitoring to prevent excessive CO_2_ absorption.^5)^ In the present series, insufflation pressure was consistently maintained at 10–12 mmHg, and EtCO_2_ was continuously monitored throughout all procedures with anesthesiologist collaboration. These precautions are considered essential to minimizing the risk of fetal acidosis in the clinical setting.^5,11)^

In the present series, preoperative CT was selectively performed when US findings were equivocal and appendicitis could not be excluded on clinical and laboratory grounds alone. While CT provides superior anatomical information regarding uterine fundus position and appendiceal location—which was critical for preoperative port planning in our series—its use during pregnancy requires careful consideration of fetal radiation exposure. The estimated fetal radiation dose from an abdominopelvic CT is approximately 8–49 mGy, which remains below the threshold generally associated with teratogenicity or significant fetal harm (approximately 100 mGy); however, this does not imply that exposure is without risk, and the principle of keeping exposure as low as reasonably achievable should always be applied. Current SAGES guidelines recommend limiting CT to situations where US or MRI does not provide sufficient diagnostic information and where the clinical need is established. In our practice, MRI was considered as an alternative when gestational age and clinical urgency permitted, consistent with the principle that the imaging modality should be individualized to the clinical context.

### Intraoperative positioning strategies

As gestational age advances, the enlarging uterus increasingly restricts the operative field and limits laparoscopic maneuverability. Left lateral decubitus positioning is generally recommended to decompress the inferior vena cava from uterine compression and to improve exposure of the pelvis and right lower abdomen.^12)^ In the present series, cases at ≥21 weeks of gestation (Cases 1 and 5) were placed in the left lateral decubitus position using a triangular lumbar pillow, and individual port placement was planned based on preoperative identification of the uterine fundus position by CT. In Case 5 (33 weeks of gestation), the left lateral decubitus position (**Fig. 1A**) combined with CT-guided port placement based on uterine fundus marking (**Fig. 1B**) provided adequate displacement of the uterus and bowel, allowing clear visualization of the uterus above the umbilicus (**Fig. 2A**) and uncomplicated identification and elevation of the appendix (**Fig. 2B**). Furthermore, coaxial trocar placement enabled mesoappendix dissection (**Fig. 3A**) and appendiceal base ligation (**Fig. 3B**) without instrument-to-uterus interference throughout the procedure. Chung et al. reported that individualized patient positioning and port placement are important determinants of technical success in LA during pregnancy and that gestational age–specific adaptation of these factors contributes to procedural safety.^13)^ Our experience is consistent with this concept; individualized preoperative port planning based on CT-confirmed fundal position was instrumental in avoiding uterine injury and ensuring a satisfactory operative field. Notably, Case 5 achieved the shortest operative time in the present series (64 min) despite representing the most advanced gestational age, suggesting that systematic preoperative planning may help offset the technical challenges associated with advanced pregnancy.

### Obstetric complications

Surgical intervention for acute appendicitis during pregnancy is widely considered justified, as the risks of perforation and peritonitis from delayed treatment substantially outweigh the perioperative risks.^3)^ Seok et al. reported, in a retrospective cohort of cases performed entirely laparoscopically, that obstetric adverse events were infrequent and that postoperative morbidity did not differ significantly between pregnant and nonpregnant patients.^3)^ In the present series, no obstetric complications were observed in any of the 6 cases during the 2nd and 3rd trimesters, reinforcing the safety of LA during pregnancy when standardized intraoperative precautions are followed. However, the management strategy for cases presenting with preoperative evidence of perforation or appendiceal abscess formation warrants specific consideration. In such advanced inflammatory scenarios, the greater risk of diffuse peritoneal contamination, technical difficulty in achieving adequate peritoneal lavage and drainage laparoscopically, and the potential for increased maternal and fetal morbidity may favor conversion to open surgery. Current SAGES guidelines specifically note that OA should be considered when laparoscopic peritoneal lavage and drainage are technically insufficient and that the threshold for conversion should be low in pregnant patients with complicated appendicitis.^5)^ Furthermore, for cases at ≥35 weeks of gestation or in other high-risk late 3rd-trimester situations, preoperative multidisciplinary consultation with an obstetrician—covering the choice of surgical approach, anesthetic management, and perioperative obstetric care—is strongly recommended before proceeding with surgery.^5,6,14)^

### Limitations

The present study has several limitations. First, the small sample size of 6 cases precludes any statistical assessment of obstetric complication rates or meaningful comparison between surgical approaches. Second, as a retrospective single-center study, selection bias cannot be excluded; the well-established multidisciplinary collaboration between surgery and obstetrics at our institution may have contributed to the favorable outcomes observed. Third, and importantly, delivery outcomes were unavailable for 3 of 6 patients (Cases 2, 3, and 4), as these patients were transferred back to their referring obstetric institutions for continued antenatal care, and no postoperative follow-up data were available from our records. This represents a significant constraint on the completeness of our obstetric safety assessment; fetal outcomes are central to evaluating the safety of surgery during pregnancy, and the absence of these data in half of the cases limits the strength of our conclusions. We were unable to obtain these data retrospectively, and this limitation should be borne in mind when interpreting the obstetric safety outcomes presented. Fourth, no cases in the1st trimester were included in the present series; accordingly, the applicability of the present findings to 1st-trimester pregnancies cannot be assessed. Fifth, neonatal outcome data, including birth weight and Apgar scores, were not systematically collected and could not be reported. These limitations preclude generalization of the present findings, and future multicenter prospective studies are warranted to validate the safety and efficacy of LA during pregnancy in the Japanese population.

## CONCLUSIONS

In this single-center case series of 6 pregnant patients during the 2nd and 3rd trimesters, gestational age–adapted perioperative management—incorporating individualized patient positioning and CT-guided port placement—facilitated safe and complete LA in all cases without obstetric complications. These findings support LA as a feasible and safe surgical option for acute appendicitis during pregnancy when appropriate preoperative planning and multidisciplinary collaboration are in place.

## DECLARATIONS

### Availability of data and materials

The datasets analyzed in this study are available from the corresponding author upon reasonable request.
